# Supplementary material for: AKAPs-PKA disruptors increase AQP2 activity independently of vasopressin in a model of nephrogenic diabetes insipidus
Source: Nat Commun. 2018 Apr 12;9:1411. doi: 10.1038/s41467-018-03771-2 (PMC5897355; doi:10.1038/s41467-018-03771-2)
Supplement: Supplementary file 1 — Supplementary Information [file 41467_2018_3771_MOESM1_ESM.pdf]

Supplementary Information

Title: AKAPs-PKA disruptors increase AQP2 activity independently of vasopressin in a model of nephrogenic diabetes insipidus

Ando et al.

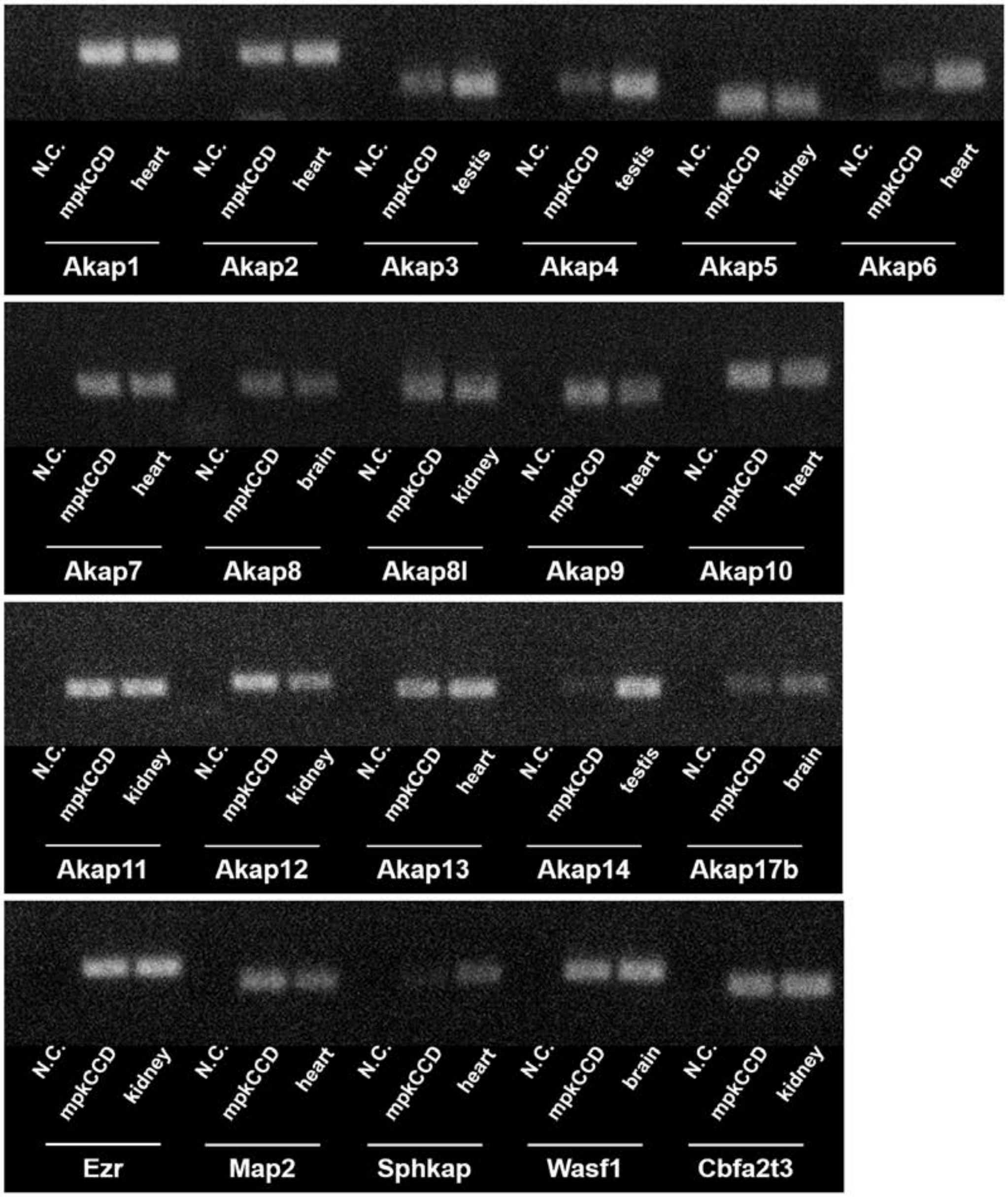

Supplementary Figure 1. Representative endogenous AKAPs are expressed in the mpkCCD cells. mRNA was extracted from the mpkCCD cells and was treated with DNase before RT-PCR assessment. N.C. indicates negative control (water in place of cDNA); cDNA from heart, testis, kidney, and brain of C57BL/6 mouse are used as positive control.

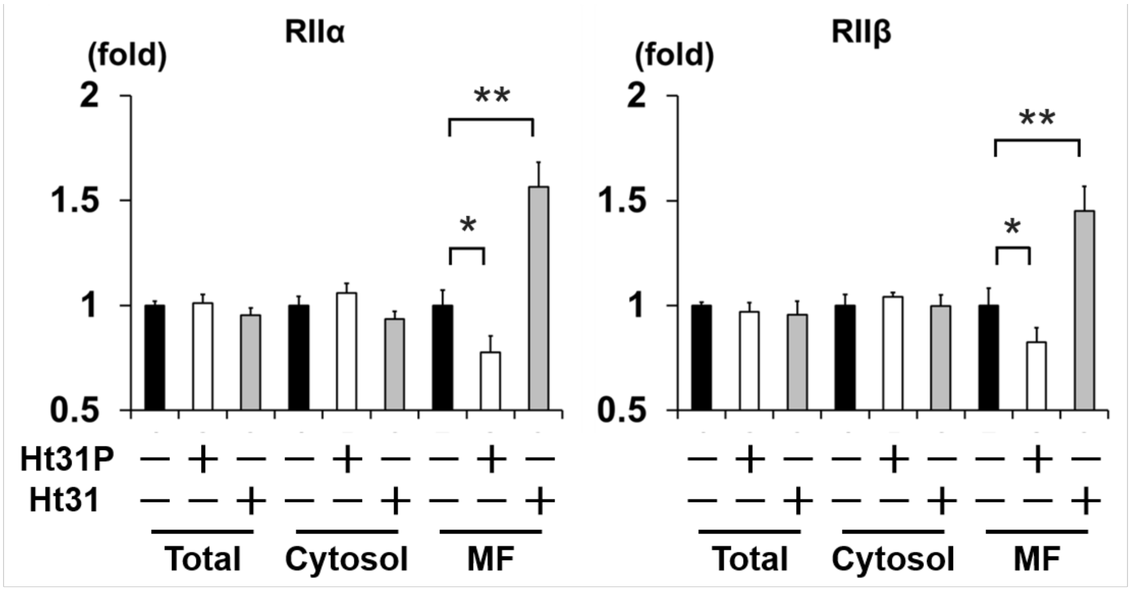

Supplementary Figure 2. Ht31 increases protein expression of PKA RIIα/β in the membrane fraction. Densitometric analysis of Figure 1e. Bands of PKA RII α/β were quantified by densitometric analysis, and the results are presented in the bar graphs as the fold change, as compared with the values of the control cells. Bars are mean values ± SD of three experiments. Tukey, *$p < 0.05$, **$p < 0.01$.

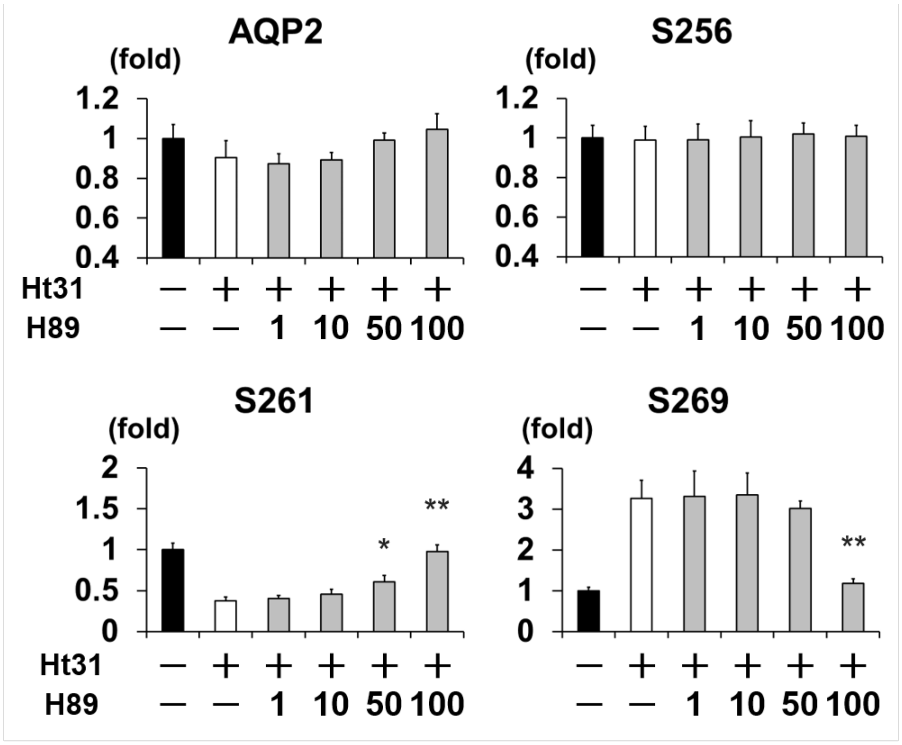

Supplementary Figure 3. Ht31 alters AQP2 phosphorylation at S261 and S269. Densitometric analysis of Figure 2b. Non-glycosylated AQP2 bands were quantified by densitometric analysis and the results are presented in the bar graphs as the fold change, as compared to the values of the control cells. Bars are mean values ± SD of three experiments. Asterisk indicates a significant difference, as compared with Ht31-treated cells. Tukey, *$p < 0.05$, **$p < 0.01$.

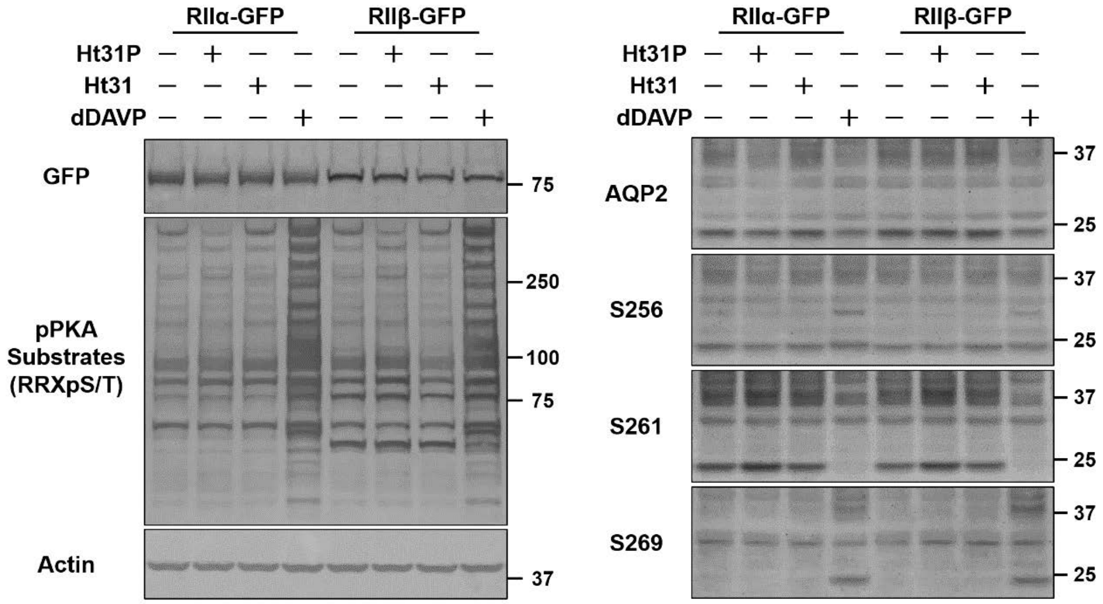

Supplementary Figure 4. Overexpression of PKA RIIα/β inhibits the Ht31-induced phosphorylation of PKA substrates and AQP2. Stable cell lines that express PKA RIIα-GFP or PKA RIIβ-GFP were generated. Ht31P (50 μM), Ht31 (50 μM), or dDAVP (1nM) was added to the basolateral side of the mpkCCD cells for 1 h. Representative blots of three independent experiments are shown.

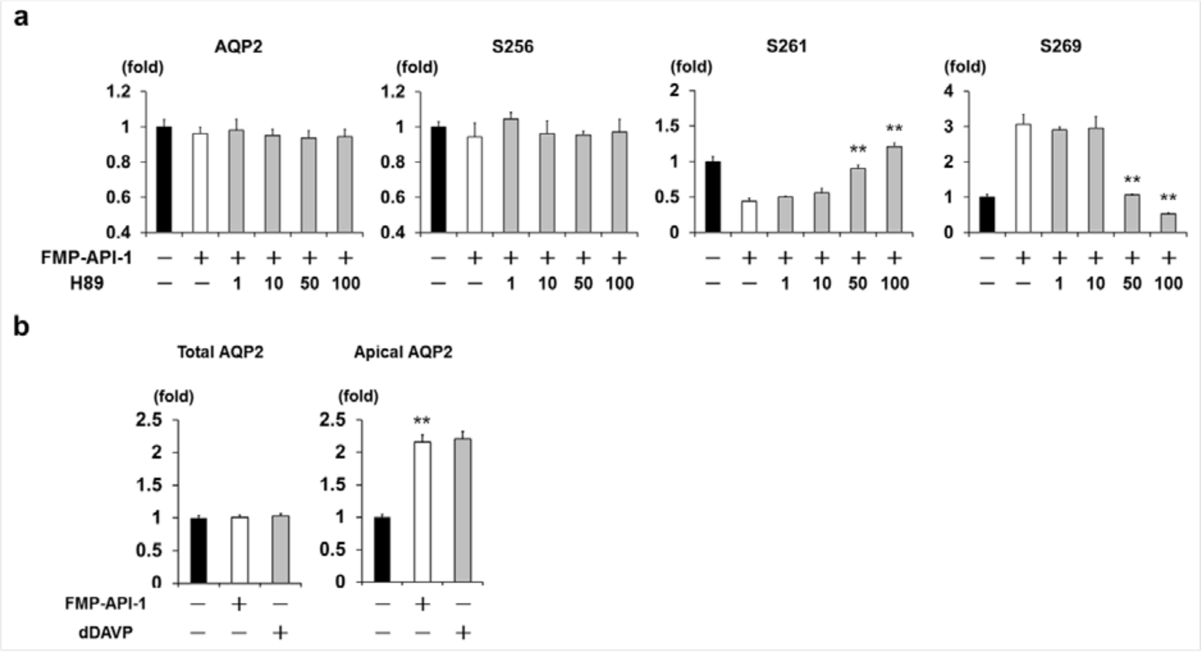

Supplementary Figure 5. FMP-API-1 alters AQP2 phosphorylation and increases apical AQP2 expression. (**a**) Densitometric analysis of Figure 4b. Non-glycosylated AQP2 bands were quantified by densitometric analysis, and the results are presented in the bar graphs as the fold change, as compared to the values of the control cells. Bars are mean values ± SD of three experiments. Asterisk indicates a significant difference compared with FMP-API-1-treated cells. Tukey, $^{**}p < 0.01$. (**b**) Densitometric analysis of Figure 4d. Non-glycosylated total and apical (biotinylated) AQP2 bands were quantified by densitometric analysis, and the results are presented in the bar graphs as fold change, as compared to the values of the control cells. Bars are mean values ± SD of three experiments. Asterisk indicates a significant difference compared with control. Tukey, $^{**}p < 0.01$.

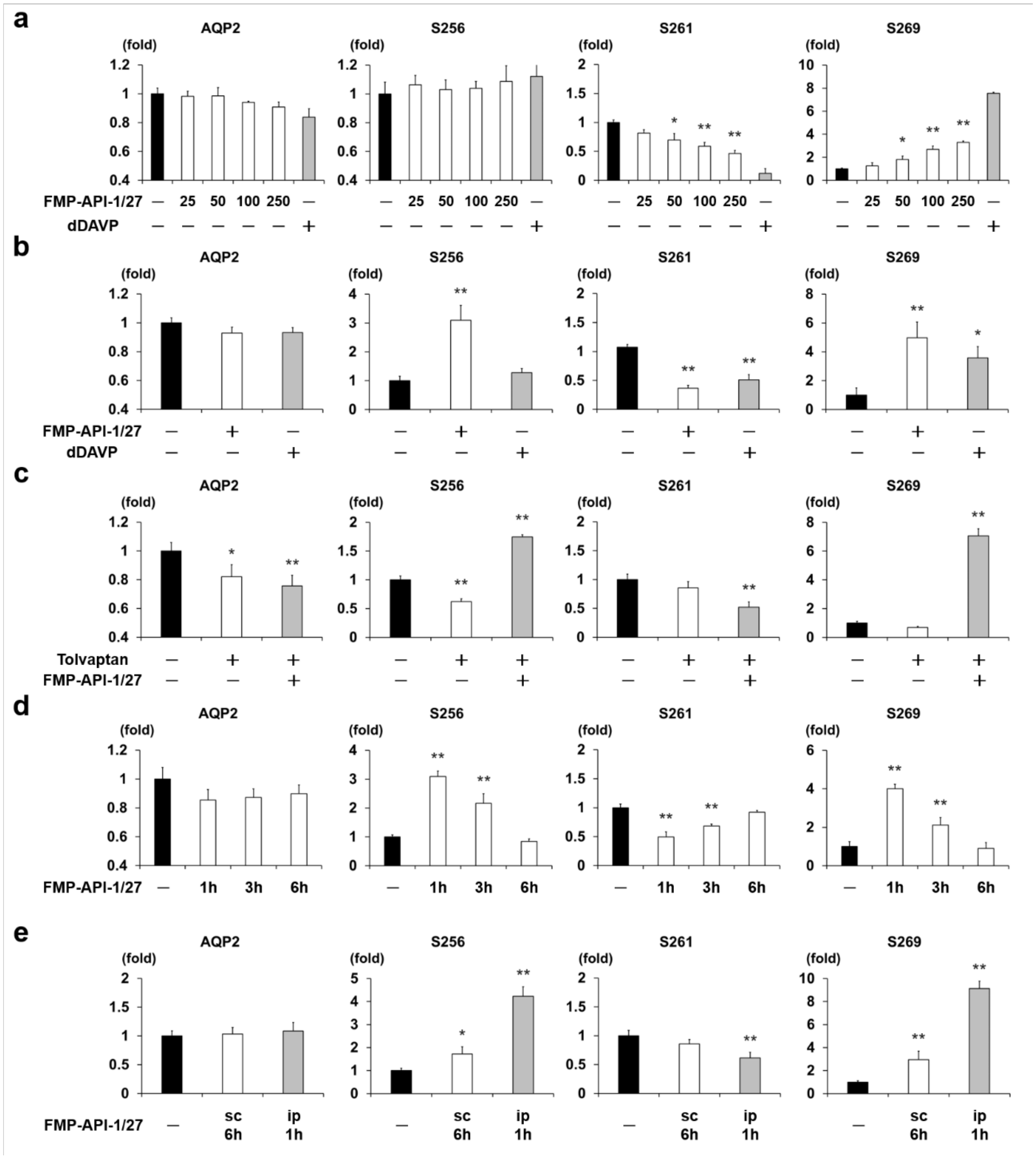

Supplementary Figure 6. FMP-API-1/27 robustly activates AQP2. (**a**) Densitometric analysis of Figure 5b. Non-glycosylated AQP2 bands were quantified by densitometric analysis, and the results are presented in the bar graphs as the fold change, as compared to

the values of the control cells. Bars are mean values ± SD of three experiments. Tukey, *p < 0.05, **p < 0.01. (**b-e**) Densitometric analysis of Figure 5c,d,f. Non-glycosylated AQP2 bands were quantified by densitometric analysis, and the results are presented in the bar graphs as the fold change, as compared to the values of the control mouse kidneys. Bars are mean values ± SD of four experiments. Tukey, *p < 0.05, **p < 0.01.

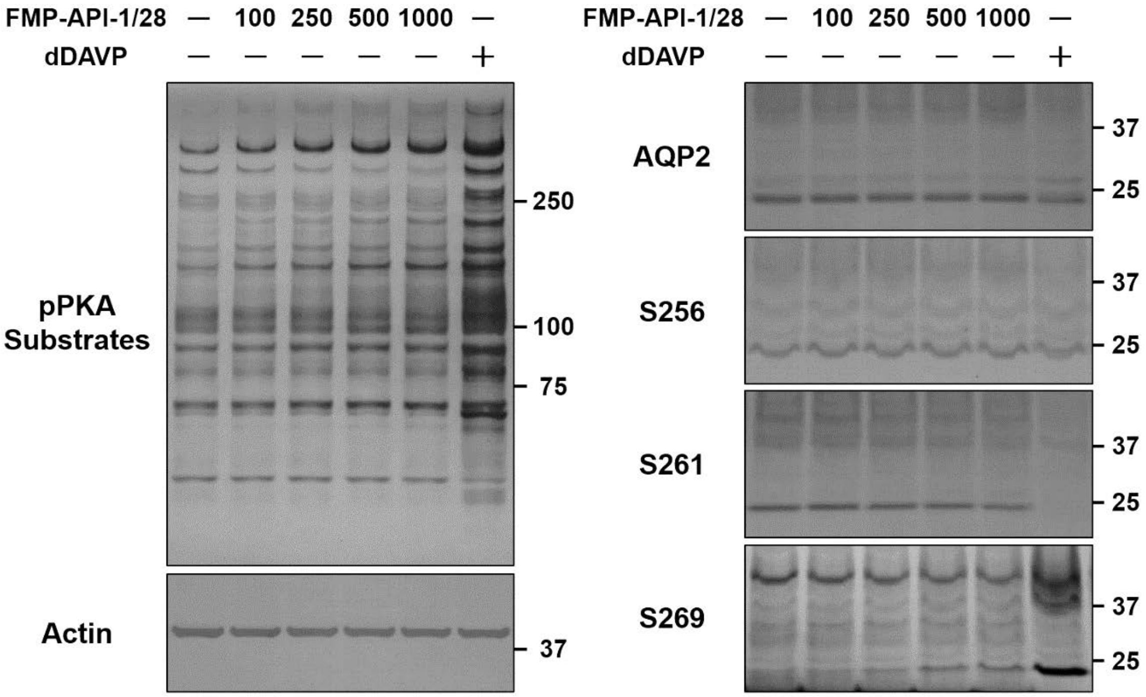

Supplementary Figure 7. FMP-API-1/28 dose-dependently induces phosphorylation of PKA substrates and AQP2. FMP-API-1/28 (100–1000 μM) was added to the basolateral side of the mpkCCD cells for 1 h. Representative blots of three independent experiments are shown.

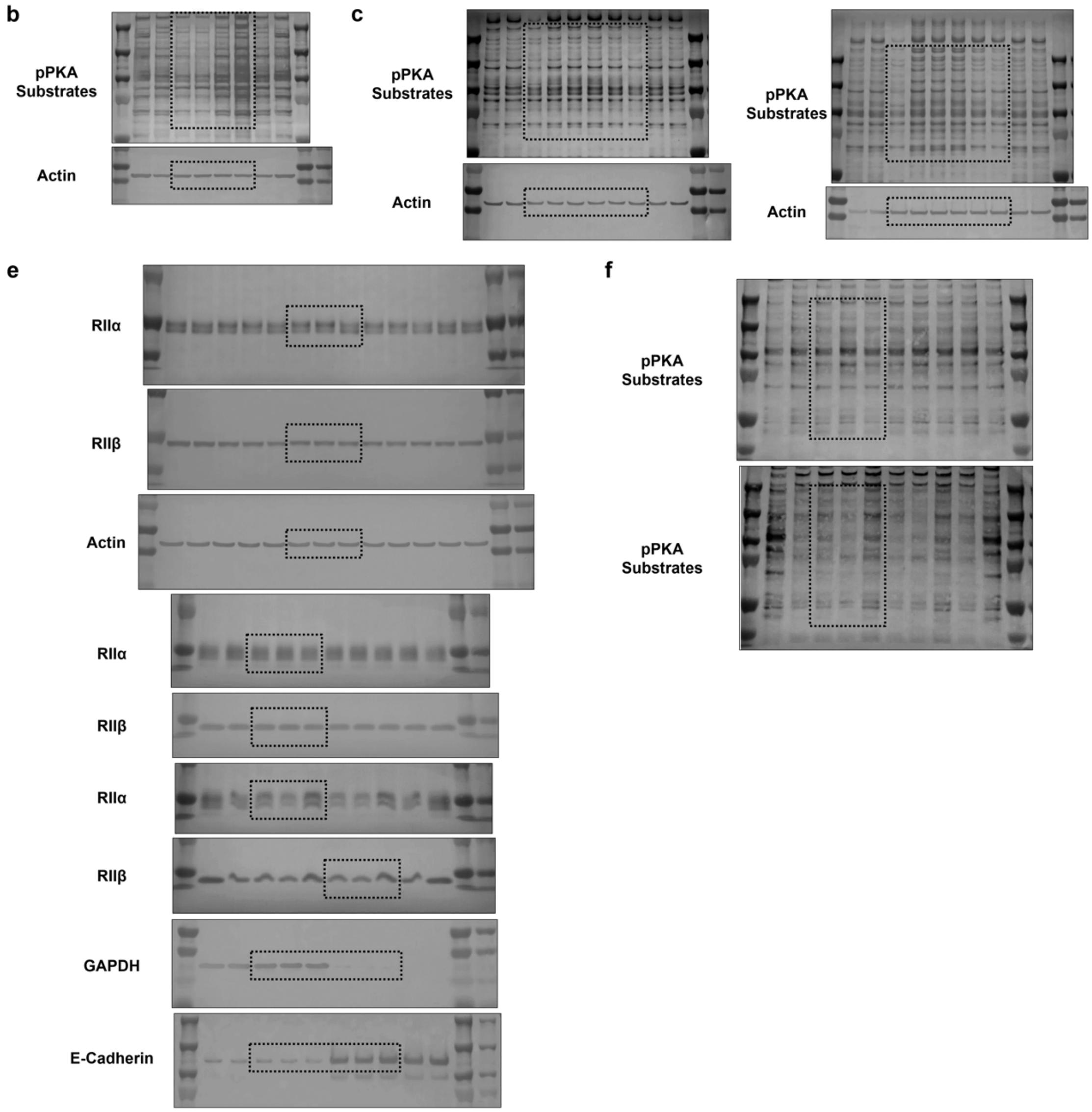

Supplementary Figure 8. Uncropped western blots of Figure 1.

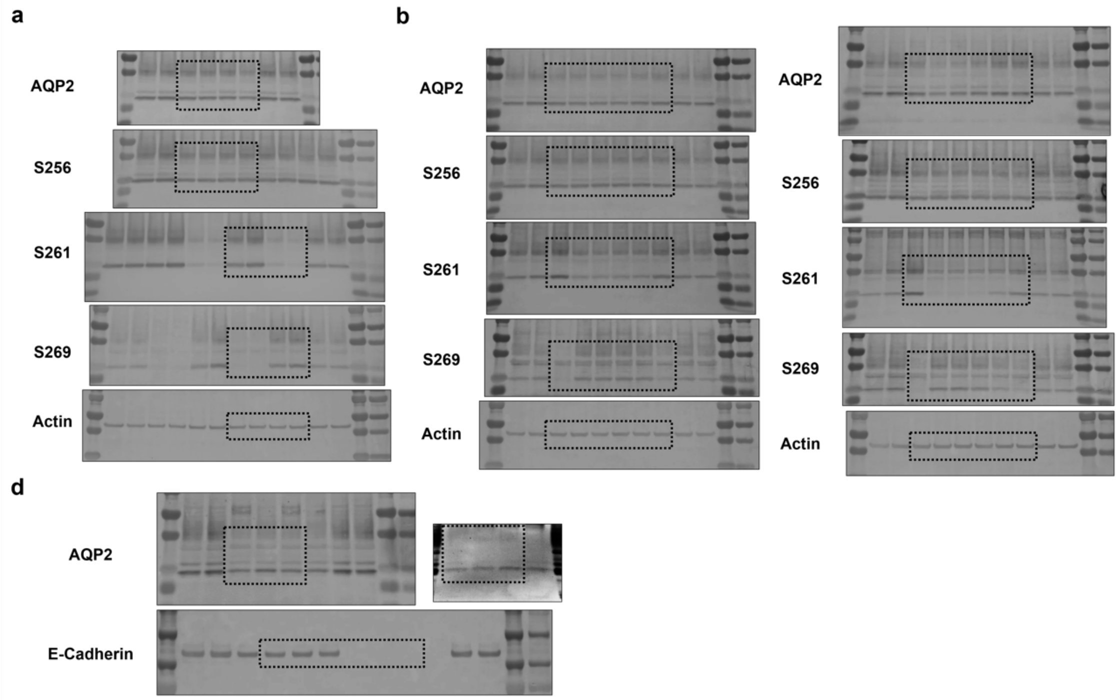

Supplementary Figure 9. Uncropped western blots of Figure 2.

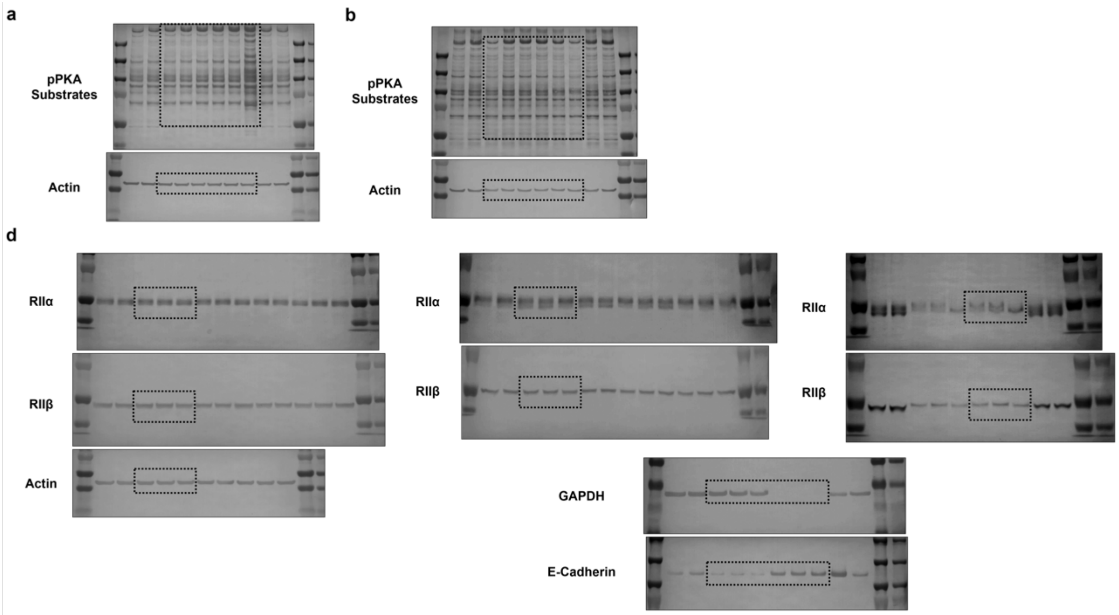

Supplementary Figure 10. Uncropped western blots of Figure 3.

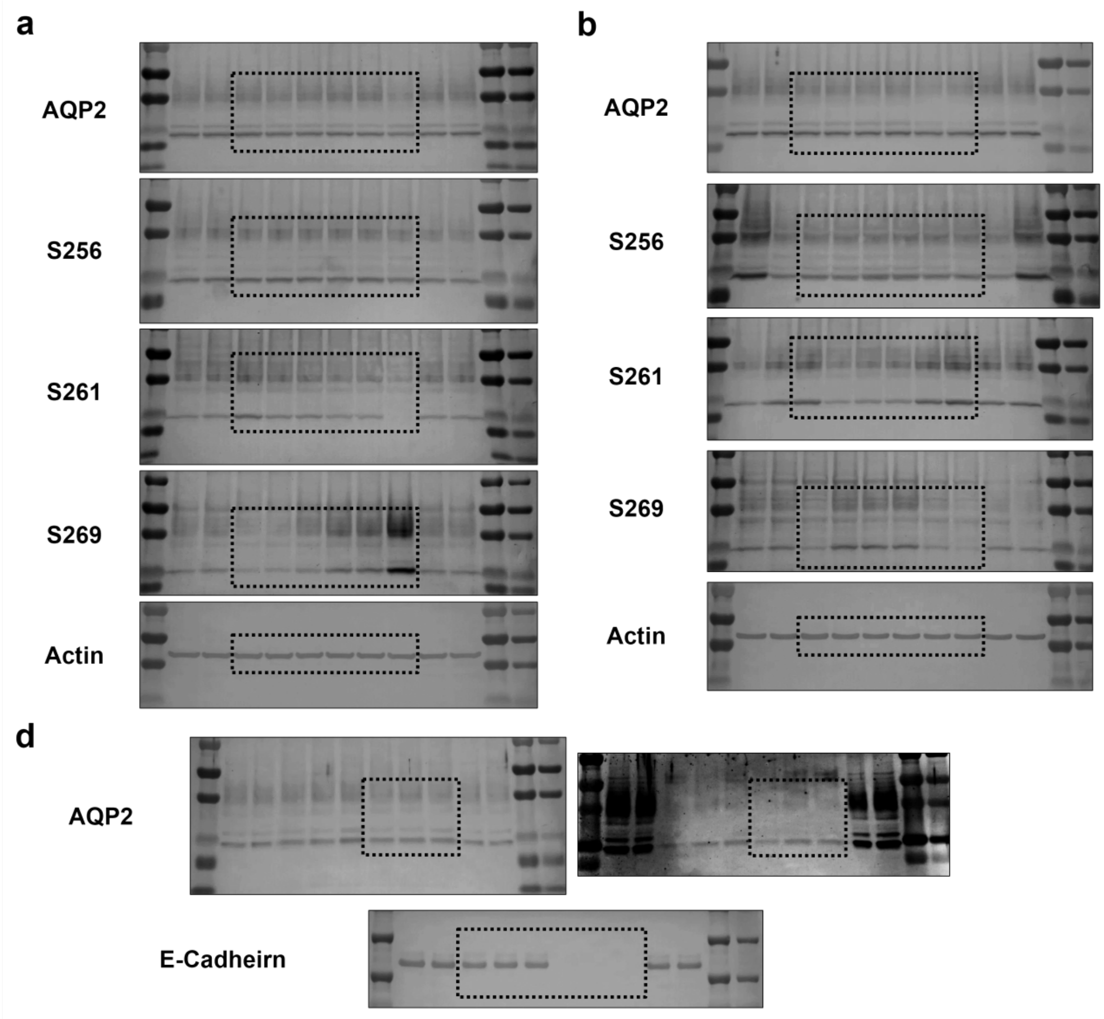

Supplementary Figure 11. Uncropped western blots of Figure 4.

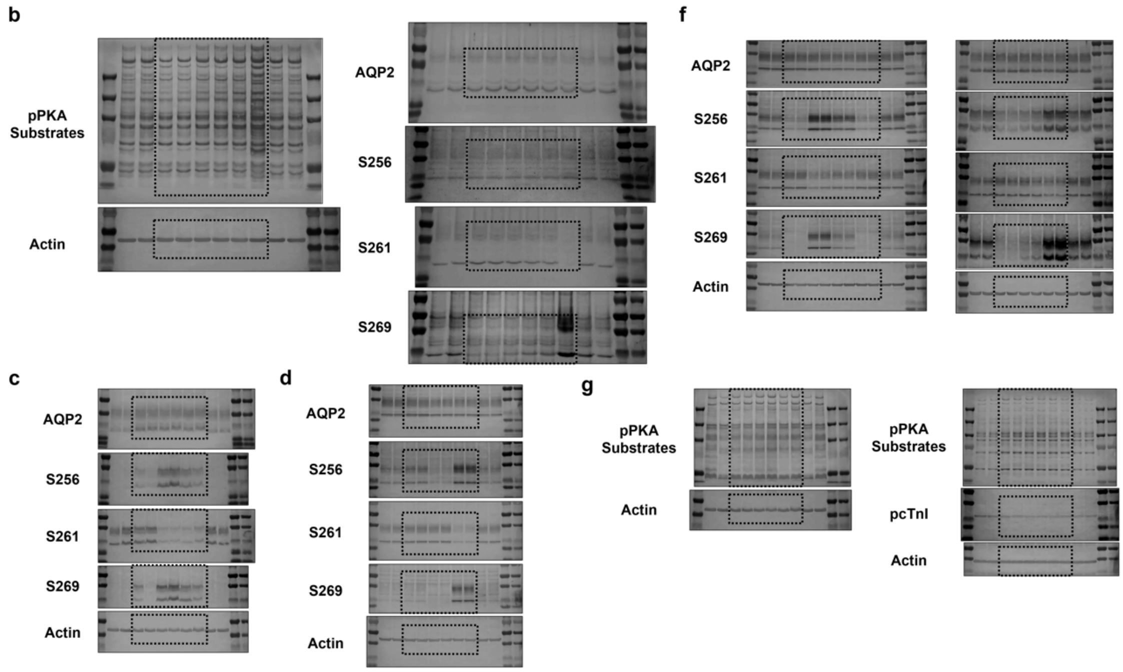

Supplementary Figure 12. Uncropped western blots of Figure 5.

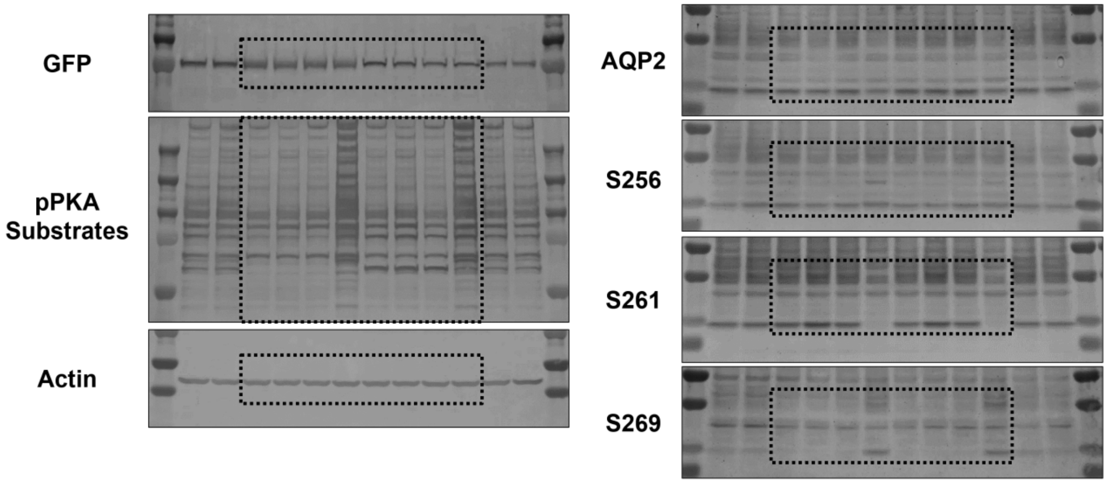

Supplementary Figure 13. Uncropped western blots of Supplementary Figure 4.

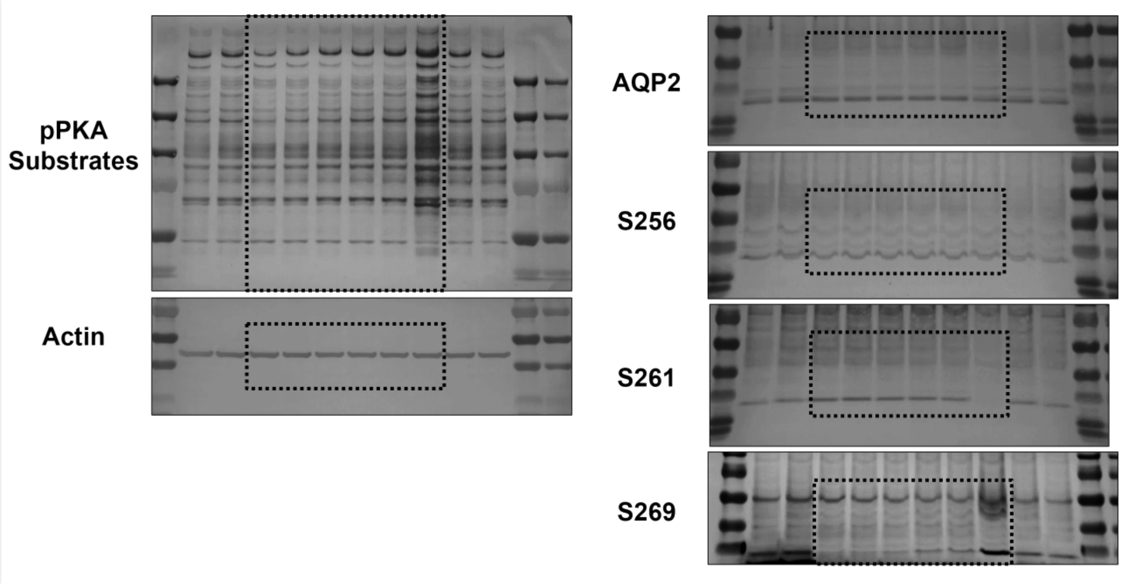

Supplementary Figure 14. Uncropped western blots of Supplementary Figure 7.

Supplementary Table 1. Primer sequences

Gene	Forward primer (5′ →3′)	Reverse primer (5′ →3′)
Akap1	AGA AGC AGA TCA CTC AGG AG	TGG ATG TTC TGT GTG TAA GG
Akap2	GAG TCT GAT GTC ATG GTT GG	TTC AGT TGT GGG GGA TGG
Akap3	TTT GTA CTT TGC TGG TGA CG	CAT TTC CCA CTG CTT CAT CC
Akap4	GGA GAC TCT GGA AGA AAA AG	TTC TGG AGA TCA TTG AGG AG
Akap5	AGA TTC AGG TAG AGA CGA AG	TCG CTT TCT TCC TTC TCT TG
Akap6	AGA TAC TGG CTA CTG ATG TG	TTC TTT CCT CTT GCT ACC AC
Akap7	CCA TTC GTT GAG GAG ATC C	CTA GGA TGC CTT TTT CCC G
Akap8	TCA GGA GAT GAA GAA TTC AG	CAT ACA GAA CAG GCA AAC TG
Akap8l	AGA AGG CAA AGA AGA TTC GG	AGA GAG AAC ACA CAA ACT GG
Akap9	GGA AAG TTC TCA GAG GGT AG	AAA TTC TTC TTC CTC CGA GC
Akap10	GAT ATC CTC TTC TGT GAG TC	CTT GGA GGG AAA AGT ACT TG
Akap11	GGA GTA GTC ACC TAA AGA GC	CTG GTT TAA ACA CTC TCC TC
Akap12	GGA AGA AGT CAC TGT TGA AG	CTG TTC GAT TAT TTC CGG TG
Akap13	ATT CAG CCC CAT TCC TAT TG	GAT TTT CTC TGC AGC CTT TG
Akap14	GAA ACC CCA TCA AGA ACA TC	ATC AGC ATA ATA CAC CCA GC
Akap17b	GAA GGA GCT ATA CAG AGG AG	TGG GCT TTC CTT ACT TCA TC
Ezr	TGG AAA TGT ATG GGA TCA AC	GTT GAA AGA GAT GTT CCT G
Map2	TCT CTC TGA ATA GCT CCA TC	AGT TGA TCC AGG GGT AGT AG
Sphkap	CGG TCC ATG AGT GAA TTA G	TTG ATC ACC AGT AGG CTC C
Wasf1	TCA ATA TCC TCA CCC CTT AC	CTG CTT TCT CTT TTC CTT CC
Cbfa2t3	TCG AAG AGT TTC ATG CCA AG	TGC AGC AGT GGA AGA TTA G
